# FOXQ1 promotes the osteogenic differentiation of bone mesenchymal stem cells via Wnt/β-catenin signaling by binding with ANXA2

**DOI:** 10.1186/s13287-020-01928-9

**Published:** 2020-09-17

**Authors:** Lusai Xiang, Junming Zheng, Mengdan Zhang, Tingting Ai, Bin Cai

**Affiliations:** 1 Guanghua School of Stomatology, Hospital of Stomatology, Sun Yat-sen University, Guangdong Provincial Key Laboratory of Stomatology, No. 56 Lingyuan west Road, Guangzhou, 510055 Guangdong China; 2 Foshan Stomatological Hospital, School of Stomatology and Medicine, Foshan University, No. 5, Hebin road, Chancheng district, Foshan, 528000 Guangdong China

**Keywords:** Forkhead box Q1, Bone mesenchymal stem cells, Osteogenic differentiation, Wnt/β-catenin, Annexin A2

## Abstract

**Background:**

This study investigated the role of Forkhead box Q1 (FOXQ1) in the osteogenic differentiation of bone mesenchymal stem cells.

**Methods:**

Mouse bone mesenchymal stem cells (mBMSCs) were transfected with lentivirus to generate *Foxq1*-overexpressing mBMSCs, *Foxq1*-suppressed mBMSCs, and mBMSC controls. The activity of osteogenic differentiation was evaluated with alizarin red staining, alkaline phosphatase activity assay, and RT-qPCR. Wnt/β-catenin signaling activities were compared among groups by TOPFlash/FOPFlash assay, immunofluorescence staining, and western blot assay of beta-catenin (CTNNB1). Coimmunoprecipitation mass spectrometry was also carried out to identify proteins binding with FOXQ1.

**Results:**

Our data showed that FOXQ1 expression was positively correlated with the osteogenic differentiation of the mBMSCs. FOXQ1 also promoted the nuclear translocation of CTNNB1 in the mBMSCs, enhancing Wnt/β-catenin signaling, which was also shown to be essential for the osteogenic differentiation-promoting effect of FOXQ1 in the mBMSCs. Annexin A2 (ANXA2) was bound with FOXQ1, and its depletion reversed the promoting effect of FOXQ1 on Wnt/β-catenin signaling.

**Conclusion:**

These results showed that FOXQ1 binds with ANXA2, promoting Wnt/β-catenin signaling in bone mesenchymal stem cells, which subsequently promotes osteogenic differentiation.

## Background

Mesenchymal stem cells constitute a group of multipotent cells capable of differentiating into various types of cells, including osteoblasts, adipocytes, and various other types of cells. This process is modulated by various signaling pathways, including BMP [1], TGF-β [2], and the Wnt/β-catenin [3] pathway. The Wnt/β-catenin signaling pathway plays an important role in mesenchymal stem cell stimulation and differentiation regulation [4, 5]. The main event in Wnt/β-catenin signaling involves the stabilization and nuclear translocation of beta-catenin (CTNNB1), which then forms a complex with TCF/LEF and initiates downstream gene transcription [6]. Similar to other major signaling pathways, Wnt/β-catenin is regulated by multiple factors [7].

Forkhead box Q1 (FOXQ1, also known as HFH1) is a member of the forkhead box (FOX) family of proteins. It was first identified as a regulator of hair follicle development [8]. Later identified as an oncogene, FOXQ1 is highly expressed in colorectal cancer, breast cancer, liver cancer, and various other cancers [9], and multiple studies have demonstrated a close relationship between FOXQ1 and Wnt/β-catenin in cancer cells [10, 11]. FOXQ1 also regulates various physiological processes, including the survival [12] and proliferation of stem cells [13]; however, its role in osteogenic differentiation remains to be elucidated.

In the current study, we aimed to investigate the influence of FOXQ1 on the osteogenic differentiation of mesenchymal stem cells and to elucidate the underlying molecular mechanisms. Our findings suggested that FOXQ1 promotes osteogenic differentiation of mouse bone mesenchymal stem cells via the Wnt/β-catenin signaling pathway.

## Methods

### Animal study and ethical approval of the protocol

To obtain mouse embryo and alveolar bone tissue for histological evaluation, pregnant Chinese Kunming (KM) mice (4 weeks old) and Chinese Kunming mice (7 days old) were purchased from Sun Yat-sen University. Chinese Kunming mice (7 days old) came from the same pregnant mouse to minimize the genetic difference between groups. The study protocol was approved by the Ethics Committee of the Hospital of Stomatology, Sun Yat-sen University (ERC-2013-15; Guangzhou, China).

### Tissue preparation and histology evaluation

To observe FOXQ1 expression in alveolar bone tissue, a pregnant KM mouse was sacrificed to obtain 3 mouse embryos at embryonic day 15.5 (E15.5), and 3 KM mice were sacrificed at each time point (postnatal day 7 (P7) and postnatal day 11 (P11)), whose mandibles were isolated surgically. The whole embryos of E15.5 mouse and the mandibles from P7 and P11 mice were fixed with 4% paraformaldehyde at room temperature for 72 h. Then, the samples were dehydrated with graded solutions of alcohol and embedded. Anti-FOXQ1 polyclonal antibody (5 μg/mL; MBS9408074; My BioSource, Inc., San Diego, USA) was used as the primary antibody.

Immunofluorescence analyses were carried out to evaluate the transnucleation of CTNNB1. Cells were incubated overnight with anti-CTNNB1 polyclonal antibody (5 μg/mL; ab2365; Abcam, Cambridge, UK) at 4 °C. Then, the sections were incubated with secondary antibody (1:1000 dilution; A-21206; Invitrogen, CA, USA) for 1 h in a dark chamber. Finally, the sections were counterstained with 4′6-diamidino-2-phenylindole (DAPI; 0.5 μg/mL; Thermo Fisher Scientific, MA, USA) for 15 min for nuclear labeling.

### Cell culture

Commercially available mouse bone mesenchymal stem cells (mBMSCs) derived from the bone marrow of Balb/c mice (MUCMX-01001; Cyagen Biosciences; Guangdong, China) were purchased and cultured with alpha-modified Eagle medium (⍺-MEM; Life Technologies, CA, USA) supplemented with 10% fetal bovine serum (FBS, Life Technologies, CA, USA), 100 U/mL penicillin (Sigma, MO, USA), and 100 mg/mL streptomycin (Sigma, MO, USA). After the 3rd passage, the cells were used for experiments.

### FOXQ1 expression manipulation

Full-length *Foxq1* cDNA was amplified with Flag-tagged primers from total RNA and then cloned into a pCDNA3.1 vector (V79020, Thermo Fisher Scientific, MA, USA) to produce the bait, Flag-tagged FOXQ1 protein, for the coimmunoprecipitation study. Full-length *Foxq1* cDNA was also cloned from total RNA and inserted into a pGLV5 vector (GenePharma, Shanghai, China). PGLV3 lentivirus containing the *Foxq1* shRNA sequence and lentivirus particles with an empty pGLV3 vector and with a pGLV5 plasmid were purchased from GenePharma (GenePharma, Shanghai, China). MBMSCs were then transfected with lentiviral particles containing *Foxq1*-sh pGLV3, *Foxq1*-overexpressing pGLV5, an empty pGLV3, or an empty pGLV5 vector, creating 4 groups of cells denoted as *Foxq1*-sh mBMSCs, *Foxq1*-over mBMSCs, lv3 mBMSCs, and lv5 mBMSCs, respectively. Real-time quantitative reverse transcription-polymerase chain reaction (RT-qPCR) and western blot assay were carried out to assess *Foxq1* expression in each group of mBMSCs.

### Osteogenic differentiation, alizarin red staining, and alkaline phosphatase activity assay

Mouse bone mesenchymal stem cells were seeded into 6-well plates at a density of 1.0 × 10^6^ cells per well. An osteogenic induction medium was prepared according to previous studies [14]. The cells were cultured in the induction medium for 14 days, during which period the medium was changed every 3 days, and then evaluated.

Alizarin red staining (ARS) was conducted to visualize the mineral deposition in each group after osteogenic induction. The cells were first fixed with cold methanol at room temperature for 10 min, rinsed twice with deionized water, and stained with alizarin red (10 μL/mL, 130-22-3; Sigma, MO, USA) at room temperature for 30 min. Then, the excess dye was removed with deionized water. For quantification of ARS, stain was desorbed with 10% cetylpyridinium chloride (CPC) in PBS, pH 7.0, for 15 min at room temperature. Then, ARS concentration was determined by absorbance at 560 nm with a spectrometer.

Alkaline phosphatase activity assay was also carried out to evaluate the osteogenic differentiation of mBMSCs from 4 groups. Cultured in the osteogenic induction medium for 7 days, 4 groups of cells were then lysed with 200 μL 1% Triton X-100. Cell lysates were then measured for alkaline phosphatase (ALP) activity with ALP measuring kit (P0321S; Beyotime, Shanghai, China). The ALP activity was measured at 405-nm wavelength with a spectrometer.

### Quantitative reverse transcription-polymerase chain reaction (RT-qPCR)

Total RNA was extracted from 4 groups of mBMSCs using TRIzol (Invitrogen, NY, USA) according to the manufacturer’s protocol. Complimentary DNA synthesis was performed with random 6-mer primers using a PrimeScript 1st Strand cDNA synthesis kit (6110A; TaKaRa Bio, Shiga, Japan). Messenger RNA expression was measured by RT-qPCR using SYBR® Green.

Various markers indicating activities of mesenchymal stem cell osteogenic differentiation were evaluated. Also, to investigate the effect of counterregulating Wnt signaling on FOXQ1’s osteogenic differentiation-promoting action in mBMSCs, Dickkopf WNT signaling pathway inhibitor 1 (DKK1, 200 ng/mL; 5897-DK; R & D Systems, Minneapolis, USA) was used to suppress Wnt signaling in the *Foxq1*-overexpressing mBMSCs, while 6-bromoindirubin-3′-oxime (6BIO, 2 μg/mL; 3194; R & D Systems, Minneapolis, USA) was used to activate Wnt signaling in the *Foxq1*-sh mBMSCs. The mRNA levels of *Wnt1*, *Wnt3*, and *Wnt10a* were then evaluated with RT-qPCR. The relative fold change for the expression of the target gene was normalized to the level of glyceraldehyde-3-phosphate dehydrogenase (*Gapdh*). The primers employed are listed in Table 1.

**Table 1:** Primer for RT-qPCR analysis

Gene	Sequence
*Foxq1*	Forward 5′-ACTGATGACAGCAGAACGCA-3′
	Reverse 5′-AGGTGTATTCGCTGTTGGGG-3′
*Alp*	Forward 5′-GCCCTCTCCAAGACATATA-3′
	Reverse 5′-CCATGATCACGTCGATATCC-3′
*Ocn*	Forward 5′-TTGTGCTGGGGTGGTTTCTG-3′
	Reverse 5′-AGCCTTCCCCAACCCCTATT-3′
*Opg*	Forward 5′-CTCCTGGACATCATTGAATGGAC-3′
	Reverse 5′-AGTTTCTGGGTCATAATGCAAGT-3′
*Runx2*	Forward 5′-GCACAAACATGGCCAGATTCA-3′
	Reverse 5′-AAGCCATGGTGCCCGTTAG-3′
*Wnt1*	Forward 5′-CCCAGGGTTCATAGCGATCC-3′
	Reverse 5′-TAGGGACCCGAGAGACAAGG-3′
*Wnt3*	Forward 5′-TCCAACTATTGGGGGCGTC-3′
	Reverse 5′-TTCATAGCTGAGCGGGCATC-3′
*Wnt10a*	Forward 5′-CTGAACACCCGGCCATACTT-3′
	Reverse 5′-GCTGTAAGAGCCAACCACCT-3′
*Gapdh*	Forward 5′-CTACCCCCAATGTGTCCGTC-3′
	Reverse 5′-GGGATAGGGCCTCTCTTGCT-3′

### Western blot analysis

*Foxq1*-sh mBMSCs, *Foxq1*-over mBMSCs, lv3 mBMSCs, and lv5 mBMSCs were seeded at a density of 1 × 10^6^ cells per well. Total proteins were extracted using RIPA buffer following the manufacturer’s protocol. Nuclear protein was isolated with a nuclear protein extraction kit according to the manufacturer’s instructions (78833; Thermo Fisher Scientific, MA, USA). The primary antibody was anti-CTNNB1 (1 μg/mL; ab2365; Abcam, Cambridge, UK) and anti-FOXQ1 (0.5 μg/mL; sc-166265; Santa Cruz Biotechnology, Dallas, USA). Anti-THOC1 (2 μg/mL; ab487; Abcam, Cambridge, UK), anti-EEF1A1 (1 μl/mL; ab140632; Abcam, Cambridge, UK), and anti-GAPDH (1 μl/mL; ab9485; Abcam, Cambridge, UK) were used as controls for the nuclear proteins, cytoplasmic proteins, and total proteins, respectively. All assays were performed in triplicate. Quantitative analysis of western blot assays was carried out with ImageJ software [15].

### TOPFlash/FOPFlash reporter assay

To assess the influence of FOXQ1 levels on Wnt/β-catenin signaling activities, a TOPFlash/FOPFlash reporter assay was performed. Cells from the *Foxq1*-sh mBMSC, *Foxq1*-over mBMSC, lv3 mBMSC, and lv5 mBMSC groups and *Foxq1*-over+si*Anxa2* mBMSCs were seeded on 96-well plates at a density of 4 × 10^3^ cells per well. Then, they were transiently transfected with TOPFlash or FOPFlash luciferase reporter plasmid (17-285; Millipore Sigma; MA, USA) according to the manufacturer’s protocol. The firefly luciferase activity level was normalized against the Renilla luciferase activity level. The fold increase indicating the TOPFlash activity compared to the FOPFlash is reported.

### Coimmunoprecipitation

To investigate the potential mechanism of the interaction between FOXQ1 and Wnt/β-catenin and subsequent signaling, coimmunoprecipitation was performed and the resultant data analyzed. *Foxq1*-overexpressing mBMSCs and lv5 mBMSCs were transfected with the Flag-tagged *Foxq1* pCDNA3.1 vector. Total protein was obtained from cell lysates from each group of cells. Coimmunoprecipitation (co-Ip) was carried out with a Pierce co-IP kit (26149; ThermoFisher Scientific, MA, USA) following the manufacturer’s protocol. In brief, 10 μg of anti-FLAG antibody (F3165, Sigma, MA, USA) was first incubated with coupling resin. The antibody-conjugated resin was then incubated overnight with 200 μL mBMSC total protein lysates at 4 °C. Then, the resin was washed, and protein complexes bound to the anti-FLAG antibody were eluted. A western blot analysis was subsequently performed as described above to confirm that the anti-FLAG antibody pulled down FOXQ1 proteins.

### Liquid chromatography–mass spectrometry and data analysis

Liquid chromatography–mass spectrometry (LC-MS) analysis was conducted with a NanoLC 400 system, and a TripleTOF 5600-Plus (AB Sciex, Toronto, Canada) system was used for the mass spectrometry (MS) analysis. ProteinPilot software (AB Sciex, Toronto, Canada) was used to analyze data from the TripleTOF 5600-Plus and identified proteins bound to FOXQ1.

The potential interactions among identified proteins were evaluated with the STRING pathway database [16]. Specifically, we tried to identify proteins associated with the Wnt/β-catenin pathway, and the proteins that were identified through the use of the STRING database were then ranked by their percentage of coverage in the LC-MS/MS results.

### Statistical analysis

Upon confirmation of a normal distribution of data, all the quantitative data were subjected to Student’s *t* tests (comparison between two groups) or one-way ANOVA, and Dunnett’s test was used as a post hoc test (comparison between 3 or more groups). *P* < 0.05 was considered significant. Statistical analyses were carried out using R 3.4.2 (R Foundation, Vienna, Austria).

## Result

### FOXQ1 expression was associated with osteogenic differentiation of mesenchymal cells in alveolar bone tissue

**Fig. 1:**
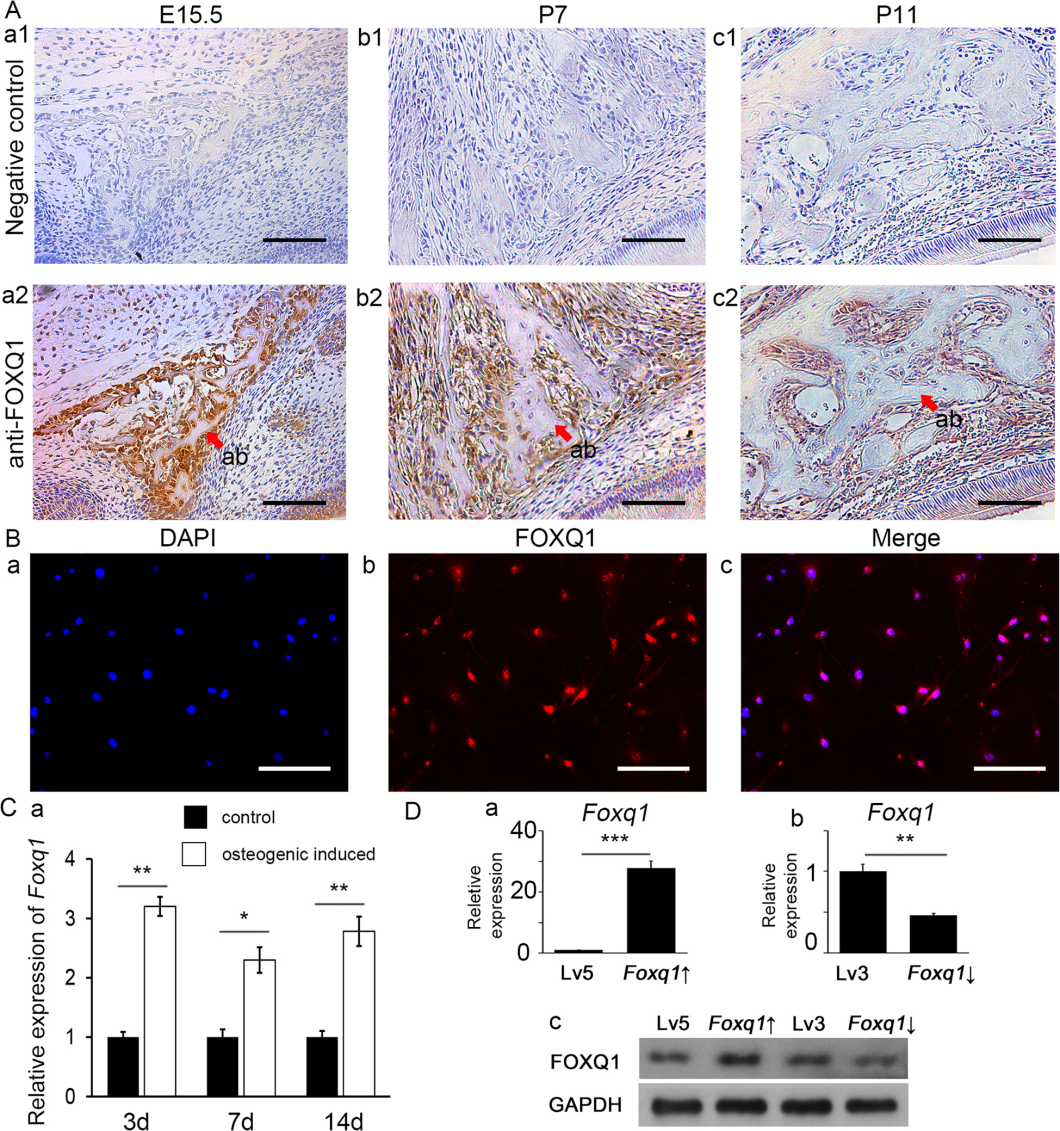
FOXQ1 was upregulated in osteogenesis. **A** Immunohistology assay results showing FOXQ1 expression in mouse alveolar bone at E15.5 (a1, a2), P7 (b1, b2), and P11 (c1, c2), scale bar 200 μm. **B** Immunofluorescence staining showed FOXQ1 in mouse bone mesenchymal stem cells after osteogenic induction, scale bar 200 μm. **C** Relative expression of *Foxq1* in mouse bone mesenchymal stem cells increased after osteogenic induction. **D** RT-qPCR results of *Foxq1* expression in Lv5 mBMSCs versus *Foxq1*-over mBMSCs (a) and Lv3 mBMSCs versus *Foxq1*-sh mBMSCs (b) and Western blot assay of FOXQ1 levels (c) confirmed manipulation of FOXQ1 expression was achieved through lentivirus transfection of mouse bone mesenchymal stem cells (Lv5, mBMSCs with empty pGLV5 vector control; Lv3, mBMSCs with empty pGLV3 vector control; *Foxq1* ↑, mBMSCs with *Foxq1* overexpressing vector; *Foxq1* ↓, mBMSCs with *Foxq1* shRNA expressing vector). *P < 0.05, **P < 0.01, ***P < 0.001

Immunohistochemical staining demonstrated a high level of FOXQ1 in alveolar bone tissue from the E15.5 mouse embryos (Fig. 1A (a1, a2)). The FOXQ1 protein level was also high in the bone tissue of the P7 mice (Fig. 1A (b1, b2)) and the P11 mice (Fig. 1A (c1, c2)) but was lower than that in the E15.5. Immunofluorescence demonstrated a high level of FOXQ1 protein in mouse bone mesenchymal stem cells (mBMSCs), and the increased protein was mainly concentrated in the cell nucleus (Fig. 1B). Furthermore, RT-qPCR showed a sustained *Foxq1* expression increase in the mBMSCs after osteogenic induction treatment (Fig. 1C).

To further study the role of FOXQ1 in the osteogenic differentiation of bone mesenchymal stem cells, FOXQ1 expression was manipulated with a lentivirus plasmid. The RT-qPCR results showed that the *Foxq1* mRNA level in the *Foxq1*-over mBMSC group was 28-fold greater than that in the lv5 mBMSC group (Fig. 1D (a)), while that of the *Foxq1*-sh mBMSC group was one-half that of the lv3 mBMSC groups (Fig. 1D (b)). These results were further validated with western blot analysis (Fig. 1D (c)).

### Increased FOXQ1 levels promote osteogenic differentiation of mouse bone mesenchymal stem cells

**Fig. 2:**
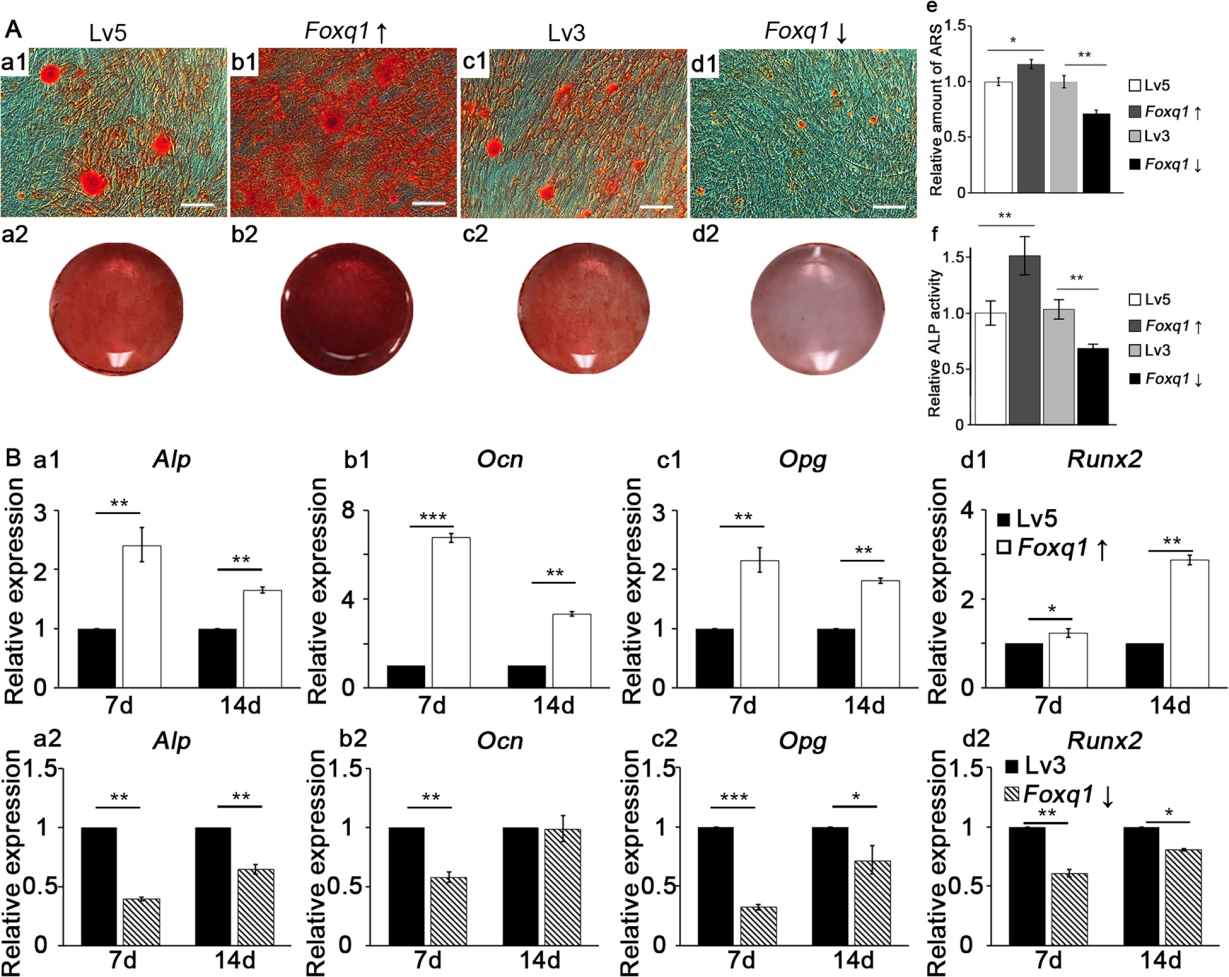
Relationship between the expression level of FOXQ1 and the osteogenic differentiation of mouse bone mesenchymal stem cells. **A** Alizarin red staining of mouse bone mesenchymal stem cells treated with osteogenic induction medium for 14 days (microscopic views: a1–d1, scale bar 200 μm; gross views: a2–d2) and the relative amount of staining (e) showed FOXQ1 expression level influence osteogenic differentiation of mouse bone mesenchymal stem cells. ALP activity assay (f) of mouse bone mesenchymal stem cells treated with osteogenic induction medium for 7 days showed a similar result. **B** Relative expression of *Alp*, *Ocn*, *Opg*, and *Runx2* in mouse bone mesenchymal stem cells at different levels of FOXQ1, 7 days and 14 days after osteogenic induction. Lv5, mBMSCs with empty pGLV5 vector control; Lv3, mBMSCs with empty pGLV3 vector control; *Foxq1* ↑, mBMSCs with *Foxq1* overexpressing vector; *Foxq1* ↓, mBMSCs with *Foxq1* shRNA expressing vector. *P < 0.05, **P < 0.01, ***P < 0.001

Seven days after osteogenic induction, ALP activity assay showed that FOXQ1 expression level was associated with ALP level in mBMSCs (Fig. 2A (f)). After 14 days of osteogenic induction, ARS showed that with increased FOXQ1 levels, mineral deposition was more prominent (Fig. 2A (a, b)), and a decrease in FOXQ1 also inhibited mineral deposition (Fig. 2A (c–d)). The quantification analysis further supported this finding. Compared to the control group, the relative amount of ARS in the *Foxq1*-overexpressing mBMSCs was 116%, and it was 71% in the *Foxq1*-sh mBMSCs (Fig. 2A (e)).

We then investigated whether FOXQ1 is able to influence the expression of osteogenesis-related molecules with RT-qPCR. On the 7th day and 14th day after osteogenic induction, various osteogenic markers, namely, *Alp* (Fig. 2B (a1)), *Ocn* (Fig. 2B (b1)), *Opg* (Fig. 2B (c1)), and *Runx2* (Fig. 2B (d1)), were upregulated in the *Foxq1*-overexpressing mBMSC group and downregulated in the *Foxq1*-sh mBMSC group, compared to the levels of the control groups. These results demonstrated a dose-dependent relationship between FOXQ1 levels and osteogenic marker expression in response to the osteogenic induction.

### FOXQ1 promotes osteogenic differentiation via Wnt/β-catenin signaling

**Fig. 3:**
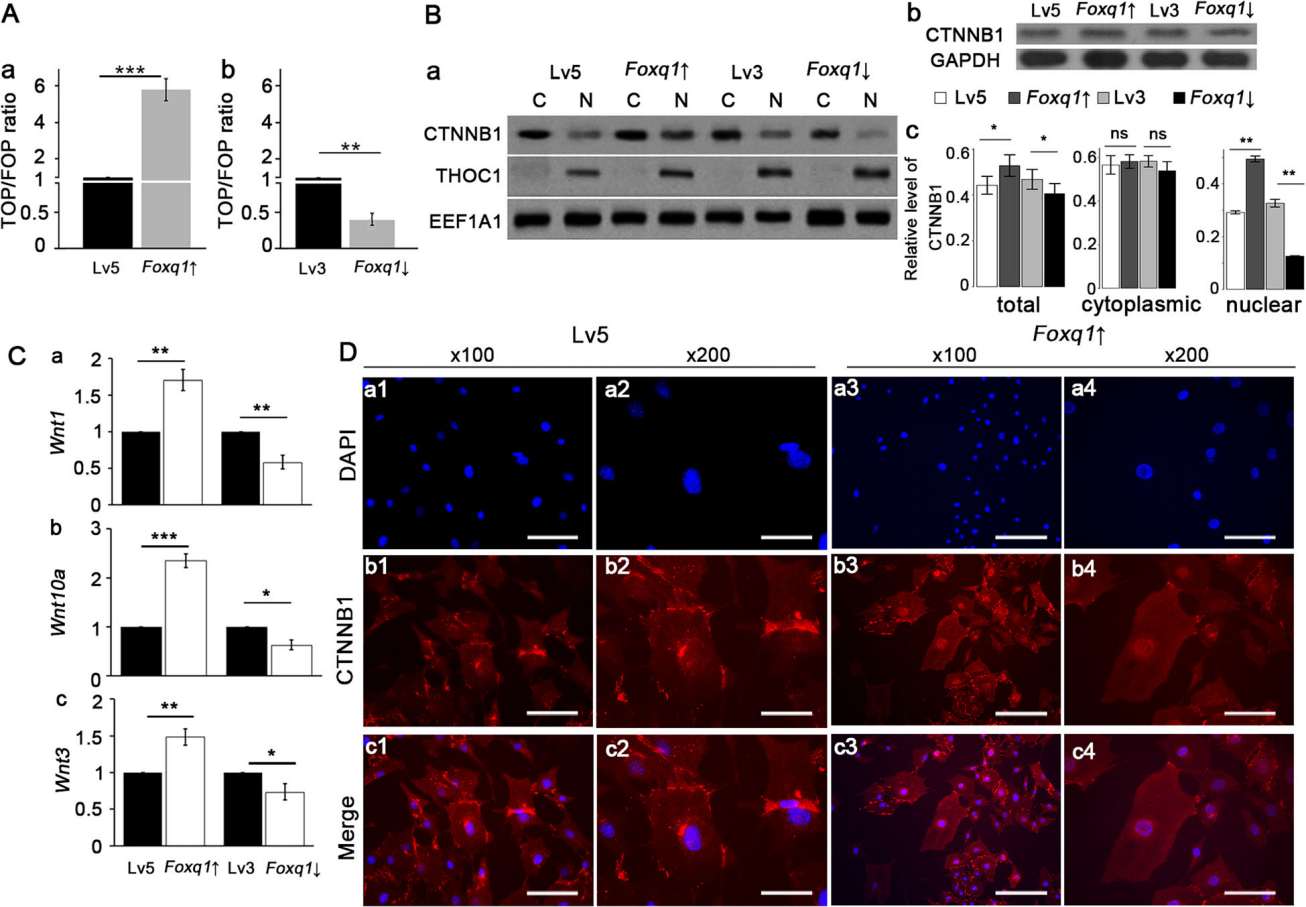
FOXQ1 promoted Wnt/β-catenin signaling. **a** TOP/FOP ratio of mouse bone mesenchymal stem cells expressing different levels of FOXQ1. **b** Western blot of nuclear and cytoplasmic beta-catenin (CTNNB1) levels in cells with FOXQ1 overexpressed and inhibited (a, C: cytoplasmic, N: nuclear, THOC1 serve as the loading control for nuclear protein, EEF1A1 serve as the loading control for both nuclear and cytoplasmic protein). Total CTNNB1 levels were also compared among groups of mouse bone mesenchymal stem cells (b); relative level of CTNNB1 normalized to loading control was evaluated for total, cytoplasmic, and nuclear CTNNB1(c). **c** RT-qPCR of *Wnt1* (a), *Wnt10a* (b), and *Wnt3* (c) on FOXQ1 expression. **d** CTNNB1 transnucleation by immunofluorescence. Scale bar for a1, b1, c1, a3, b3, c3, 200 μm; scale bar for a2, b2, c2, a4, b4, c4, 100 μm. Lv5, mBMSCs with empty pGLV5 vector control; Lv3, mBMSCs with empty pGLV3 vector control; *Foxq1* ↑, mBMSCs with *Foxq1* overexpressing vector; *Foxq1* ↓, mBMSCs with *Foxq1* shRNA expressing vector

Wnt/β-catenin is an important pathway in the regulation of osteogenic differentiation. The TOPFlash/FOPFlash assay of β-catenin/TCF/LEF transcriptional activity demonstrated a 5.7-fold increase in the *Foxq1*-over mBMSCs (Fig. 3A (a)). For the *Foxq1*-sh mBMSCs, the transcriptional activity was one-half that of the lv3 mBMSCs (Fig. 3A (b)). The subcellular fractional western blot analysis showed that, while the levels of cytoplasmic β-catenin (CTNNB1) protein were similar across the groups (Fig. 3B), a clear dose-dependent relationship was established between FOXQ1 and intranuclear CTNNB1 (Fig. 3B (a, c)). Western blot analysis comparing total CTNNB1 across the groups (Fig. 3B (b, c)) also showed a dose-dependent relationship with FOXQ1, but less prominent than that of intranuclear CTNNB1. These results were further supported by an immunofluorescence study showing an increase in the overlap between DAPI and CTNNB1 in *Foxq1*-overexpressing mBMSCs, demonstrating an increase in the nuclear translocation of CTNNB1 (Fig. 3D).

*Wnt1*, *Wnt3*, and *Wnt10a* are indicators of Wnt/β-catenin activity level, and their expression levels were evaluated with RT-qPCR. The overexpression of FOXQ1 upregulated *Wnt1* (Fig. 3C (b)), *Wnt3* (Fig. 3C (c)), and *Wnt10* (Fig. 3C (d)) expression, while FOXQ1 suppression led to the inhibition of their expression (Fig. 3C (b, c, d)).

**Fig. 4:**
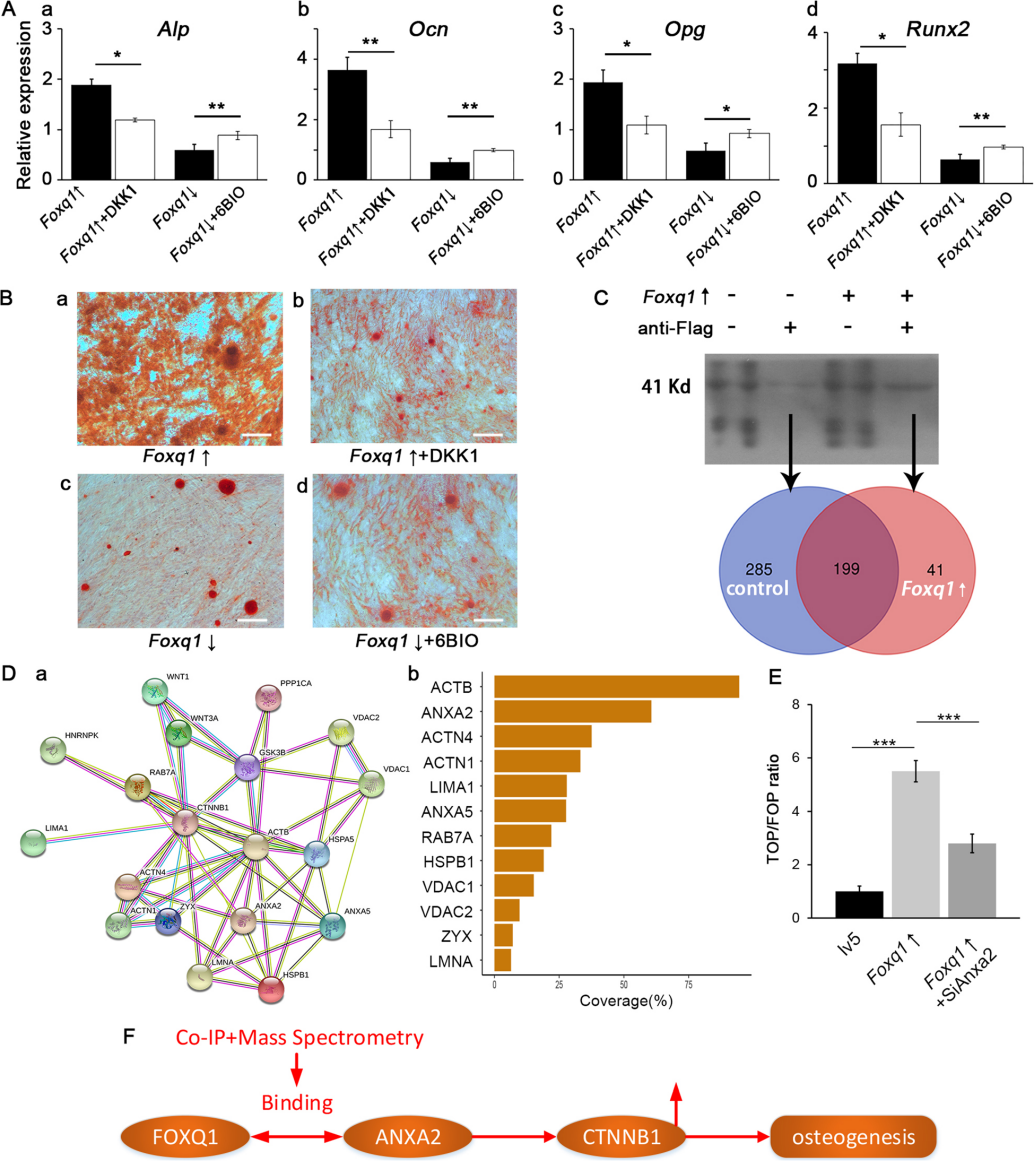
FOXQ1 promoted Wnt/β-catenin signaling by interacting with ANXA2. **a** RT-qPCR of *Alp* (a), *Ocn* (b), *Opg* (c), and *Runx2* (d) showed altered Wnt/β-catenin signaling pathway activity with DKK1, and 6BIO reversed the effect of FOXQ1 overexpression and inhibition. **b** Alizarin red staining of mouse bone mesenchymal stem cells showed altered Wnt/β-catenin signaling pathway activity with DKK1, and 6BIO reversed the effect of changed FOXQ1 expression on osteogenic differentiation. Scale bar 200 μm. **c** Co-Ip/MS identified 199 proteins bound with FOXQ1 in both control and *Foxq1*-overexpressing mouse bone mesenchymal stem cells. **d** Protein-protein interaction network (a) constructed by the STRING online database based on 15 matches, and these proteins were ranked by their coverage percentage (b) in the Co-Ip/MS results. **e** The TOP/FOP ratio of mouse bone mesenchymal stem cells showed that depletion of *Anxa2* mRNA reversed the promotion of FOXQ1 on Wnt/β-catenin signaling. **F** Schematic representation illustrates FOXQ1 binding to ANXA2, which increases the transnucleation of CTNNB1, promoting Wnt/β-catenin signaling and osteogenesis

The treatment with DKK-1 attenuated the FOXQ1-induced increase in osteogenic differentiation (Fig. 4B (a, b)) and osteogenic marker expression (Fig. 4A (a–d)), while 6BIO alleviated the suppression of the osteogenic differentiation (Fig. 4B (c, d)) and expression of the related markers (Fig. 4A (a–d)).

### FOXQ1 promotes Wnt/β-catenin signaling via interaction with ANXA2

The coimmunoprecipitation and LC-MS analysis identified various proteins that were pulled down with FOXQ1. A total of 484 proteins were identified in the samples from the lv5 mBMSC group, while 240 proteins were identified in the samples from the *Foxq1*-over mBMSC group, and 199 proteins were identified in both types of samples to be binding with FOXQ1(Fig. 4C). With the STRING database, 12 proteins were found to be possibly linked to Wnt/β-catenin (Fig. 4D). Among the candidates with coverage greater than 50% (Fig. 4D (b)), ANXA2 was shown in a previous study to enhance the transnucleation of CTNNB1. The TOPFlash/FOPFlash assay also confirmed that *Anxa2* mRNA depletion with *siAnxa2* reversed the increase in the Wnt/β-catenin signaling in the *Foxq1*-overexpressing mBMSCs, suggesting that ANXA2 is an integral part of the mechanism by which FOXQ1 promotes Wnt/β-catenin signaling.

## Discussion

FOXQ1 is a member of the forkhead box family, and a recent study showed that various members of this family were involved in the osteogenesis process. FOXO1 was found to be an early molecular regulator during mesenchymal stem cell differentiation into osteoblasts [17]. FOXC1 was also demonstrated to promote osteogenesis by regulating RUNX2 during bone formation [18]. Being a transcription factor, FOXQ1 is involved in a wide variety of processes. It has been identified as an oncogenic factor in various carcinomas [19–22], and it also participated in various physiological processes [8, 12, 13], but its role in the osteogenic differentiation of mesenchymal stem cells is still unknown. In the current study, evident expression of FOXQ1 in bone tissue and mouse bone mesenchymal stem cells was demonstrated. Additionally, osteogenic induction treatment promoted FOXQ1 expression. On the other hand, FOXQ1 upregulation promoted osteogenic differentiation of mouse bone mesenchymal stem cells, while FOXQ1 suppression led to the opposite effects. These results showed that FOXQ1 is also a factor regulating the osteogenesis process.

Our results showed that FOXQ1 overexpression or suppression led to enhanced or attenuated Wnt signaling activity in mBMSCs, respectively. Wnt/β-catenin signaling is an important regulator of osteogenic differentiation [5, 23]. Beta-catenin is the central molecule in this signaling pathway; when intranuclear translocated, it would bound with TCF/LEF and promote the transcription activity of various osteogenesis associated factors [24], including OPG [25], OCN, ALP [26], and RUNX2 [27], promoting osteogenic differentiation of mesenchymal stem cells [26]. We also showed that the influence of FOXQ1 on the osteogenic differentiation of mBMSCs was partially reversed by counterregulating the activity of Wnt/β-catenin signaling, through the Wnt/β-catenin signaling suppressor DKK1 in the *Foxq1*-over mBMSC group or the Wnt/β-catenin signaling activator 6BIO in the *Foxq1*-sh mBMSC group. From these results, it can be inferred that FOXQ1 promotes the osteogenic differentiation of mesenchymal stem cells via the Wnt/β-catenin signaling pathway.

Previous studies have shown that FOXQ1 expression is mediated by Wnt/β-catenin [10]. Interestingly, FOXQ1 silencing prevents the nuclear translocation of β-catenin, reducing Wnt/β-catenin signaling [4]. But the detailed mechanism for crosstalk between them is still unclear. We try to explore the possible mechanism with co-IP LC-MS study, and ANXA2 was found to bind with FOXQ1. ANXA2 is a member of the family of calcium-dependent proteins [28] that participate in angiogenesis, ion channel activation, and intercellular interactions [29, 30]. It was also shown to be associated with increased levels of CTNNB1 [31, 32]. A previous study [33] showed that lncRNA–MUF binds ANXA2, which enhances its binding to glycogen synthase kinase 3 beta (GSK3B) and disrupts the formation of the GSK3B/CTNNB1 complex. In our study, *Anxa2* mRNA depletion reversed the promoting effect of FOXQ1 overexpression, demonstrating that binding with ANXA2 is important for FOXQ1 function in Wnt signaling regulation to promote osteogenic differentiation of mesenchymal stem cells.

## Conclusion

In summary, our study highlights the importance of FOXQ1 as a mediator of mesenchymal stem cell osteogenic differentiation. To the best of our knowledge, this is the first study to demonstrate that FOXQ1 regulates the activities of Wnt/beta-catenin signaling by binding with ANXA2. These results provide novel insights into the mechanism underlying the osteogenic differentiation of mesenchymal stem cells.

## Data Availability

The datasets used and/or analyzed during the current study are available from the corresponding author on reasonable request.

## Abbreviations

Alp: Alkaline phosphatase
ANXA2: Annexin A2
ARS: Alizarin red staining
Co-Ip: Coimmunoprecipitation
EEF1A1: Eukaryotic translation elongation factor 1 alpha 1
FOXQ1: Forkhead box Q1
Gapdh: Glyceraldehyde-3-phosphate dehydrogenase
KM mice: Chinese Kunming mice
LC-MS: Liquid chromatography–mass spectrometry
mBMSCs: Mouse bone mesenchymal stem cells
Ocn: Osteocalcin
Opg: Osteoprotegerin
Runx2: Runt-related transcription factor 2
TCF/LEF: T cell factor/lymphoid enhancer factor
THOC1: Nuclear matrix protein P84
